# The Role of Grit on Students' Academic Success in Experiential Learning Context

**DOI:** 10.3389/fpsyg.2021.774149

**Published:** 2021-10-18

**Authors:** Jiaze Li, Yang Li

**Affiliations:** ^1^School of Foreign Studies, Nanjing University, Nanjing, China; ^2^School of Administration, Nanjing Forest Police College, Nanjing, China

**Keywords:** grit, experiential learning, students' success, educational success, pedagogical implications

## Abstract

Students' success as a cognitive issue in learning is prejudiced by proper learning approaches which improve their comprehension and achievement. In an attempt to scrutinize supplementary or alternate variables that envisage students' success, the researcher inspected a non-cognitive factor, namely grit, theorized as passion and perseverance due to its long-term quality, on the one hand, and its popularity among scholars in preceding decades on the other hand. Moreover, experiential learning (EL) is a momentous instructional approach used in the educational process to accelerate “do it and learn.” The proposed review aims to gauge the EL approach as well as grit to regulate learners' educational success. Consequently, some pedagogical implications are presented for teachers, students, and syllabus designers.

## Introduction

The success of learners primarily depends on not only proficiency tests, which aim to test learning capabilities but also a limited set of academic abilities (Sternberg et al., 2012). Positive psychology (PP) is a comprehensive academic field that concentrates on elements that promote learners' success and well-being (Wang et al., 2021; Zeng, 2021) and their psychological stability by emphasizing optimal human performance (Lopez et al., 2015). To obtain knowledge of the elements that lead to achievement and success, it is critical to evaluate people at their best, following PP (Seligman, 2011; Pishghadam et al., 2021) because learners with similar capabilities and preparation may attain equivalent academic success; however, this type of success may differ greatly (Dweck et al., 2011) in that individuals' personality, intelligence quotient or effort may vary from person to person. The potential to learn across different learning areas has traditionally been linked to educational success at a variety of degrees and there has been a growing interest in this concept across a diverse range of settings. Nevertheless, educational success depends on a multitude of interrelated factors and cannot be attributed to merely one factor (Paat et al., 2020). To preserve and certify learners' success, higher education is seeking other ways to ascertain and determine it containing not only students' cognitive but also their non-cognitive traits, as well. Researchers and university administrators widely recognized that the presence of social skills, such as communication, initiative, flexibility, and perseverance, are essential for educational success (Farruggia et al., 2018) and these socio-emotional factors consist of traits or behaviors associated with engagement and academic success of college learners (Sedlacek, 2017). As a moderately new construct in the educational realm and within a PP paradigm, grit embraces theories of passion and perseverance (Farruggia et al., 2018; Mattick et al., 2021; Wang et al., 2021), and it is deemed as a non-cognitive skill that is known in predicting success (Sommerfeld, 2011; Alan et al., 2019). As stated by Duckworth et al. (2007), a learner's competence to continue after complications is known as grit and the study distinguishes a positive effect of grit on persistence, self-control, and self-guideline, and it also alludes to mental strength in endeavoring toward achievements (Reed and Jeremiah, 2017). It is proven that traits such as grit influence psychological performance through the reduction of stress, depression, and tension (Zhang et al., 2018; Mosanya, 2019), and enhancement positive feelings such as efficacy, self-regulation, pleasure, well-being, and optimism (Salles et al., 2014; Kim et al., 2018; Kim, 2019; Datu and Restubog, 2020). Learners who show energy toward their homework and continue with their project, despite scholarly and social difficulties, are probably going to encounter scholastic achievement (Allen et al., 2021). Indeed, it has been shown that teachers who encourage grit can help learners to achieve their learning goals by motivating them to try hard and persevere in this process (Huéscar Hernández et al., 2020). Gritty people not only can perform tasks but also keep track to achieve goals throughout their education progress and they are interested in learning involvement, the durability of commitment, and perseverance through stimulating teaching (Eskreis-Winkler et al., 2014). According to Stoltz (2015), every successful person has perseverance, a great attribute that executives value above any other characteristic when selecting people to achieve any notable goal. Achievement and success are considered to be the effect of both perseverance and consistency. Perseverance is a result of the first failures that an applicant faces on the way to success in a field while consistency is a result of many hours of concentrated effort (Credé et al., 2017).

One of the primary objectives of many individuals in education is looking through various educational practices that trigger learners' enthusiasm for learning and increment the learning results successfully (Balan et al., 2015). In conventional teaching, sometimes referred to as the teacher-centered method, the actual teaching takes place as the educator directs the lesson, and the learners sit passively and just listen to the educator. Moreover, conventional education regularly underlines the completion of tasks and memorization, which describe surface learning (Turner and Baskerville, 2013). In contrast, coordinating new materials with existing information is known as reflective learning (i.e., experiential instruction), which gives learners a special chance to deal with difficult abilities to recreate in a conventional class but will be needed for accomplishment in their work after graduation (Bradberry and De Maio, 2018). In the most recent decades, and as indicated by educators, EL keeps on being well-known in higher education (Barnes, 2016), and experiential, student-focused training keeps on acquiring infinite acknowledgment (Kolb, 2014). Moreover, Kolb and Kolb (2018) claimed that EL is a constructive method of learning inspired by the learner, and it intentionally seeks to link better career, college, and personal learning results together (Holmes et al., 2018). Thus, as declared by Kolb and Kolb (2005), EL can be depicted from a constructivist structure where information is made and reproduced in the student's individual information and not simply by passing on previous notions to the student (Kolb and Kolb, 2018). Through their endeavors, students build information, learn-by-doing as they participate in tackling issues, either alone or cooperatively, and critically ponder over bits of knowledge that arise (Watts et al., 2011; Che et al., 2021). Learners' involvement in solving problems in the learning system is the main contribution of EL, which is its innate characteristic. Experiential instruction can improve learners' education and workplace execution by building critical thinking abilities, problem-solving aptitude, and the capacity to deal with multifaceted problems in reality (Butler et al., 2019). EL programs give learners a special chance to deal with abilities that are difficult to recreate in a conventional class but will be needed for accomplishment in their work after graduation (Bradberry and De Maio, 2018).

Through active learning, learners can master knowledge, retain information, improve problem-solving skills, and gain cognitive flexibility (Brickner and Etter, 2008). The learning-through-experience method encourages participation, interaction, difficulty, and personal responsibility of the learning process. However, some essential elements for knowledge creation are not required for active learning and EL theory states that learning takes place when learners analyze, interpret, and make use of knowledge (McCarthy, 2010). Numerous studies examined the relationships between grit and cognitive or non-cognitive issues like educational achievement or personality traits (Ransdell, 2001). For instance, the positive connections exist between grit and grade point average (GPA) and accomplishment (Chen et al., 2015; McDermott et al., 2015), and completion of homework (Bennett et al., 2013). Correspondingly, several studies have verified the efficiency of active learning (Benecke and Bezuidenhout, 2011; Penger et al., 2011; Maskulka et al., 2012) that they proved that EL significantly influences learning purposes that may have a prolonged effect on students as they get ready for progressive educational scholarships and professional provision. Although studies on grit, success, and EL have been carried out; just some have currently carried out reviews about the variables and they have not been investigated together so far; consequently, regarding this lacuna, this review makes an effort to consider them in education.

## Grit

Grit has been introduced as a distinctive feature noticeable in successful learners (Duckworth, 2016), and it is a conception that should be regarded as both social and emotional and certain attention is paid to it concerning one's success in his life (Brooks and Seipel, 2018). Characterized as a compound and stable individual attribute, grit impacts mentalities and practices across various settings and is shared by the most exceptional innovators in each field (Wolters and Hussain, 2015). Grit can also be characterized as the enthusiasm and determination to achieve long-haul objectives, despite difficulties and afflictions, and it could be dynamic personality strength for the occasions when people experience their own difficulties and concerns or when they experience crucial circumstances (Lozano-Jiménez et al., 2021). Generally, grit incorporates the ability to sustain both the interest and exertion in projects that can require some time to finish. Those who do not steer from their original objectives have a high level of grit (Duckworth and Quinn, 2009; Tough, 2012). Thus, the essence of grit can be regarded as the follow-through or the intentional, persistent devotion to exercises and obligations experienced to accomplish one's objectives effectively (Duckworth et al., 2007). In studies of grittier people, academic and non-academic performance improved, and motivation increased as they discovered meaning in the achievement of success (Von Culin et al., 2014). A successful person with added perseverance is not only extremely motivated but also eager to concentrate on fulfilling long-term, more ambitious goals, as well as being adaptable and less concerned with daily routines (Duckworth et al., 2007; Duckworth, 2016).

## Experiential Learning

Learning by performing and dealing with problems is an important concept in experiential education that helps learners acquire knowledge, abilities, and behaviors through challenges they encounter and allows learners to become proficient by practicing and overcoming obstacles (Andreu-Andrés, 2016). Kohonen (2007) suggests that EL is a rich learning environment where learners learn through their own experiences as well as others' experiences and includes both observational and hands-on learning. An EL program may be conducted in the academic context or the workplace (Schwartz, 2015), and this form of learning is popular among informal learning contexts, like working in organizations and companies, medical knowledge, global experience, and community service. As this method explores specific aspects like abilities, tactics, and context, the learners become familiar with those particular elements which indeed enhance their learning performance (Kolb, 2014). The direct participation of learners, their dynamic commitment, and work-based learning chances have been distinguished as the vital components of EL (Mak et al., 2017). Learners engaging in this kind of learning will attain help in the improvement of their fundamental abilities, basic reasoning, critical thinking, hard-working attitude, collaboration, correspondence, and leadership abilities (Roberts, 2018). Moreover, as has been confirmed by Ruzek et al. (2014), how to adopt these integrative, limit-crossing methods of EL connecting the class with the rest of the world has the best effect on higher education. Different advantages and recipients of EL have been accounted for; however, the learner is the best recipient (Schwartz, 2015). Learners in modules taught through EL techniques are regarded to be more ready for the workforce, with better moral thinking, more significant levels of inventiveness and ingenuity, and further developed lateral and basic reasoning abilities (Clem et al., 2014). Furthermore, they demonstrated better multicultural comprehension and sensitivity and they are well-prepared to connect theory with practice, and they have greater levels of confidence and they demonstrate promoted degrees of motivation. Since teachers become more acquainted with the learners as individuals and are compelled to reconsider the course content, they take advantage of applying an EL model (Clem et al., 2014). Schwartz (2015) additionally highlighted these advantages by expressing that through EL, colleges guarantee that learners have the important abilities to outperform professionally.

In addition, learning within interactive/experiential settings also involves the application of concepts through guided in-class exercises, activities, and tasks that are accompanied by relevant examples and even illustrations (Peterson et al., 2015; Wong and Kawash, 2020). Active learning is deemed as a vital factor in experiential and student-centered instructive contexts, and engaging students in everyday and realistic situations are among the aspects of the education as providing learning activities and methods are not only effective but also motivating for learners when they are given some responsibilities during the learning process (Laguador and Dizon, 2013). As learners participate in group projects in experiential education settings, they obtain responses and reactions immediately and be conscious of the teamwork value, which helps them skip the competitive situation that often takes place when learners do not have chances to work together effectively to achieve their goals (Canziani et al., 2015). It is common for learners to experience short-term challenges or discouragement while trying to learn a new field of education or strategy for finding solutions. Generally, learners who are incapable of pushing forward despite obstacles or disappointment are less likely to succeed (Arslan et al., 2013).

## Implications and Future Directions

The current review has innumerable academic, operational, and theoretical implications. Concerning theory, the study distinctively added to the body of the current literature regarding grit by proposing introductory confirmation about its function on students' success in the EL context. This review aims to present some evidence that the grit construct may be a significant factor leading to improved educational success among learners. It is also proved that perseverance has a remarkable role in the learning process. Based on the literature review, it was found that effort and persistence as a facet of grit may impact educational success. Moreover, perseverance is more precisely relevant to educational success due to other factors, such as teaching methods. Besides, to be successful in the process of learning, educators must improve students' grit in classrooms and through extra-curricular EL activities.

In line with the literature review, the learners can benefit from this type of research by attending in a learner-centered learning context. Indeed, there should be a more profound, more critical, and longer enduring change in the manner by which the learners are instructed and supported in the class and the utilization of a learner-focused learning approach, like EL, is fortified. Since it requires the student to distinguish and find assets, foster inquiries characterize issues, create speculations, and goes through individualized assessment, EL is regarded as learner-focused (Butler et al., 2019). Learners are bound to persevere despite challenges when they are offered a proper learning strategy that assists them to be more successful in their learning and permits them to completely participate in scholastic assignments. By building learners' collection of learning systems, educators can increase learners' perseverance indirectly. In addition, students may take advantage of activities presented in EL to enhance their grit and consequently their success. Learning through experiential activity makes it simple for learners to think by themselves, to be cooperative and work in a team, gather and handle information, introduce notions, make large presentations ahead of time, and are proactively dynamic in learning. Simply put, it is an active learning technique that can be executed in many fields of information, hence articulating limits for students and cultivating learners' character successfully (Brickner and Etter, 2008). Learning by doing and applying prior learned information through EL tasks and activities is noteworthy for students as they are provided with notable prospects to enhance their professional skills, implement and increase their theoretical information, engage in classroom tasks (Lan and Moscardino, 2019; Derakhshan, 2021; Xie and Derakhshan, 2021), and improve moral traits and the resulting personal and professional growth will promote learners' success (Sternberger et al., 2005). In addition, character strengths such as grit enhance student perseverance when confronting challenges, enhance academic performance, and help graduates stay in higher education longer. For example, a large number of university students obtain their bachelor degrees when they complete their study in the universities. They may be faced up with two options—finding a job directly or pursing their further studies. It is obvious that pursuing one's further studies needs one's effort as well as perseverance. In this regard, if one cannot persist in what they have been doing, he/she hardly ever go to other institutions to pursue their further studies. Thereby, grit may play a vital role in helping graduates stay in higher education longer.

Moreover, the educators should plan teaching methods using learners' perspectives and values as a means of helping them understand what they are learning. Experiential education builds learning experiences and engages learners by allowing them to make sense of learning. In addition to facilitating EL, teachers should act as a facilitator to prepare and implement activities that trigger the experiences of the learners and encourage them to push through failure and persist with difficult tasks that also stimulate their sense of grit. It has been proved that gritty humans have better powers of identifying the solutions to demanding challenges rather than those who are passive (Lucas et al., 2015). For gritty learners, obstacles and challenges serving as opportunities for learning lead them to be more persistent and attentive in the face of difficulties, which result in success (Dweck et al., 2011). When dealing with various environmental problems and issues during problem-solving occurred in EL, they are forced to make decisions on what would be the best solution in resolving the particular situation so when executing an action, they should demonstrate perseverance of effort that is indicative of their effort, dedication, and commitment to significant tasks (McDermott et al., 2015) measured by the grit construct, which is significantly related to attentiveness to the main decisions of life (Eskreis-Winkler et al., 2014).

Educators can help learners boost and promote grit by being aware of the assignments given to the learners and the assurance of honesty that the educators demonstrate both within the classroom and at home. Through both classroom and extracurricular activities, learners can develop perseverance as it is significant for learners to possess high levels of persistence and effort to complete their educational requirements (Hwang et al., 2017). Teachers should try to assist learners to endure when experiencing difficulties, increase their educational presentation and work on learners' maintenance that is among the objectives of creating character qualities like grit in higher education. Furthermore, teachers are encouraged to increase performance-based activities by which students can have additional opportunities to make an effort to engage in activities. Moreover, teachers should also provide emotional support by praising and identifying students' efforts to actively take part in diverse tasks. Besides, teachers must evaluate the achievements of the learning process as well as plan and observe each phase of the activity, providing feedback to learners (Villarroel et al., 2020). In this way, they are involved in the learning and try to work hard to perform well on the tasks. Since the teacher acts as a facilitator instead of a presenter in EL, they assist the students to construct perceptions and connect concepts by supporting information, guiding exploration, reinforcing understanding of difficult concepts, and presenting sources that enhance their success. Furthermore, the teacher stimulates mediation of group progression (Lozano-Jiménez et al., 2021) and upshots so he/she may be supposed as an instructor or a director who offers and arranges reaction and reinforcement (Salari et al., 2018).

Additionally, syllabus designers are suggested to design instructive and emotional instructions or programs that can promote grit among students with various dispositional backgrounds. Indeed, a more student-centric learning setting should be built in an academic setting in which grit is encouraged by exchanging the mindset of the learners (Farrington et al., 2012). Through grit-related mediations, adult students do not experience educational confusion together with dropping out whereas leading them to maintenance, educational success, and enhancements in syllabuses of their course. Unfortunately, since academic mediation and instruction programs that emphasize the enhancement of grit have not been presented to date, this review presented noteworthy shreds of evidence that have possibly set the stage for future research considering the benefits of grit in the EL context and the use of syllabuses as a way of intervention at universities that embrace more constructive learning strategies and goals that lead to higher academic success (Wurdinger and Allison, 2017). Finally, more empirical research should be done in this domain and also more studies are recommended to inspect the process and development of grit and EL via longitudinal studies to detect the modifications that teachers may experience in the process of developing education. Future research should be conducted to examine the facilitating and regulating constructs and noticeable traits of gritty learners to contribute to theoretical developments of the grit and grit-augmenting mediation platforms.

## Author Contributions

YL and JL have made substantial, direct and intellectual equal contributions to the work, and approved the publication of the work in Frontiers of Psychology.

## Funding

This work was sponsored by Key Projects of Teaching Reform of Nanjing Forest Police College “The Construction of College Students' Mental Health Teaching Model from the Perspective of Positive Psychology” (Grant No.: ZD18005), Jiangsu Social Science Foundation (Grant No.: 15JYB018), and Jiangsu Universities in Philosophy and Social Science Foundation (Grant No.: 2015SJD257).

## Conflict of Interest

The authors declare that the research was conducted in the absence of any commercial or financial relationships that could be construed as a potential conflict of interest.

## Publisher's Note

All claims expressed in this article are solely those of the authors and do not necessarily represent those of their affiliated organizations, or those of the publisher, the editors and the reviewers. Any product that may be evaluated in this article, or claim that may be made by its manufacturer, is not guaranteed or endorsed by the publisher.
